# In patients with unilateral pleural effusion, restricted lung inflation is the principal predictor of increased dyspnoea

**DOI:** 10.1371/journal.pone.0202621

**Published:** 2018-10-03

**Authors:** Luke A. Garske, Kuhan Kunarajah, Paul V. Zimmerman, Lewis Adams, Ian B. Stewart

**Affiliations:** 1 Institute of Health and Biomedical Innovation, Queensland University of Technology, Brisbane, Queensland, Australia; 2 Department of Thoracic Medicine, Prince Charles Hospital, Brisbane, Queensland, Australia; 3 University of Queensland, Brisbane, Queensland, Australia; 4 Allied Health Sciences and Menzies Health Institute of Queensland, Griffith University, Gold Coast, Queensland, Australia; Vanderbilt University Medical Center, UNITED STATES

## Abstract

**Background and objective:**

The mechanism of dyspnoea associated with pleural effusion is uncertain. A cohort of patients requiring thoracoscopy for unilateral exudative effusion were investigated for associations between dyspnoea and suggested predictors: impaired ipsilateral diaphragm movement, effusion volume and restricted lung inflation.

**Methods:**

Baseline Dyspnoea Index, respiratory function, and ultrasound assessment of ipsilateral diaphragm movement were assessed prior to thoracoscopy, when effusion volume was measured. Transitional Dyspnoea Index (change from baseline) was assessed 4 and 8 weeks after thoracoscopy. Pearson product moment assessed bivariate correlations and a general linear model examined how well total lung capacity (measuring restricted lung inflation), effusion volume and impaired diaphragm movement predicted Baseline Dyspnoea Index. Un-paired t tests compared the groups with normal and impaired diaphragm movement.

**Results:**

19 patients were studied (14 malignant etiology). Total lung capacity was associated with Baseline Dyspnoea Index (r = 0.68, P = 0.003). Effusion volume (r = -0.138, P = 0.60) and diaphragm movement (P = 0.09) were not associated with Baseline Dyspnoea Index. Effusion volume was larger with impaired diaphragm movement compared to normal diaphragm movement (2.16 ±SD 0.95 vs.1.16 ±0.92 L, P = 0.009). Total lung capacity was lower with impaired diaphragm movement compared to normal diaphragm movement (65.4 ±10.3 vs 78.2 ±8.6% predicted, P = 0.011). The optimal general linear model to predict Baseline Dyspnoea Index used total lung capacity alone (adjusted R^2^ = 0.42, P = 0.003). In nine participants with controlled effusion, baseline effusion volume (r = 0.775, P = 0.014) and total lung capacity (r = -0.690, P = 0.040) were associated with Transitional Dyspnoea Index.

**Conclusions:**

Restricted lung inflation was the principal predictor of increased dyspnoea prior to thoracoscopic drainage of effusion, with no independent additional association with either effusion volume or impaired ipsilateral diaphragm movement. Restricted lung inflation may be an important determinant of the dyspnoea associated with pleural effusion.

## Introduction

Pleural effusion has an annual incidence of 0.3%, affecting 1 million patients annually in the United States alone.[1, 2] There are multiple potential causes of pleural effusion, with ~85% caused by heart failure, malignancy, or pneumonia.[1, 2] Patients with pleural effusion experience dyspnoea which impairs quality of life and impacts on daily activities.[3]

There is limited research examining mechanisms of dyspnoea with pleural effusion.[3] Pleural effusion is associated with restrictive ventilatory limitation,[4–6] and impairs the capacity of the inspiratory muscles to generate pressure.[3, 7, 8]

Drainage of pleural effusion relieves dyspnoea,[9, 10] but is associated with relatively small improvements in lung volumes.[4–7, 11] This has suggested that restricted lung inflation associated with pleural effusion may not be an important cause of dyspnoea.[3] A cohort study showed that patients with paradoxical ipsilateral diaphragm movement associated with pleural effusion had more severe dyspnoea, and greater improvement in dyspnoea after drainage of pleural effusion.[11] A larger pleural effusion may displace the diaphragm inferiorly and impair its capacity to generate pressure, which potentially may cause neuro-mechanical uncoupling.[7] Consequently, the effect of a larger pleural effusion on diaphragm function has been suggested to be a more important cause of dyspnoea than restricted lung inflation.[3] However, we are not aware of published research examining associations between dyspnoea and either lung volumes or effusion volume.

We selected a study population of patients with unilateral exudative pleural effusion requiring thoracoscopy as part of clinical care, because these patients commonly report significant dyspnea prior to drainage, and thoracoscopy allows complete removal of the effusion to determine its volume.[12] The primary study objective was to determine the associations between dyspnoea prior to thoracoscopy and the following predictors: restricted lung inflation, effusion volume, and impaired ipsilateral diaphragm movement. This objective was achieved. A secondary aim was to determine whether the same parameters predicted improvement in dyspnoea after sustained thoracoscopic control of pleural effusion. Preliminary data was obtained for this aim.

## Materials and methods

Patients with a unilateral exudative pleural effusion requiring thoracoscopy as part of routine clinical care were prospectively recruited from a tertiary hospital (Brisbane, Australia) over 2.5 years. Patients with significant alternative causes of dyspnoea, cognitive impairment or language barrier were excluded. However, patients with mild co-morbidity potentially causing dyspnoea were not excluded (see Results for details). We reasoned that including these participants would increase the generalisability of our findings, as patients with pleural effusion frequently have significant co-morbidity.[13] This study was approved by the Princess Alexandra Hospital Research Ethics Committee, and conformed to the Declaration of Helsinki. All participants provided written informed consent.

In the 24 hours before thoracoscopy, participants had a chest radiograph, baseline dyspnoea index (BDI), respiratory function, and trans-thoracic ultrasound. BDI is a score to measure the impact of dyspnoea with usual daily activities in the 2 days prior to thoracoscopy, which ranges from 0 (most severe dyspnoea) to 12 (no significant dyspnoea). Two independent interviewing clinicians completed scores for three domains: functional impairment, magnitude of task, and magnitude of effort, with the score for each domain ranging 0–4.[14] Respiratory function (spirometry, lung volumes, diffusion capacity of carbon monoxide & maximal inspiratory/expiratory pressures) was measured (62J body plethysmograph, Sensormedics, Yorba Linda, California, USA) according to guidelines,[15–18] and compared to predicted values.[19–22] Transthoracic ultrasound was performed (Logiq100, GE Medical Systems, Milwaukee, Wisconsin, USA) immediately prior to thoracoscopy. Participants were in the lateral de-cubitus position with the effusion side up, and the probe was oriented in the coronal plane in the mid-axillary line. Ipsilateral diaphragm movement with spontaneous breathing was assessed as either normal or impaired (reduced or paradoxical movement) as previously.[11, 23, 24]

Thoracoscopy was performed as part of usual clinical practice. All endoscopically visible pleural fluid was removed into a graduated cannister. Any pleural fluid which leaked out of the thoracoscopy port was added to the graduated canister to measure total effusion volume. 5 g talc was insufflated when malignant pleural effusion was suspected. An intercostal catheter was removed when drainage was <150 ml/day. A chest radiograph was repeated within 2 hours of thoracoscopy.

Chest radiograph and transitional dyspnoea index (TDI) were repeated at 4 and 8 weeks after thoracoscopy. TDI is the change from the baseline prior to thoracoscopy (BDI was not repeated) in the impact of dyspnoea with usual daily activities.[14] For both TDI assessments, participants were interviewed about the impact of dyspnoea with usual activities over the previous 2 days, while noting the examples and grades previously recorded with BDI assessment prior to thoracoscopy. Possible scores range between -9 (major deterioration) and +9 (major improvement). Two independent clinicians completed scores for the three domains: functional impairment, magnitude of task, and magnitude of effort, with the score for each domain ranging -3 to +3.

If dyspnoea index (BDI and TDI) differed between the two clinicians, a consensus score was reached after re-interviewing participants. Dyspnoea index and diaphragm movement were assessed by independent clinicians blinded to the results of respiratory function.

Two clinicians independently assessed the sequence of chest radiographs, to determine whether the pleural effusion was controlled after 4 and 8 weeks. Pre-specified criteria for control of effusion were: less than 50% re-accumulation of fluid compared to baseline chest radiograph, and no further drainage required.[13] Assessment of control differed between the two clinicians on one occasion, when a consensus decision was reached after reviewing chest radiographs together.

### Statistical analysis

Statistical analysis was performed using SPSS statistical package, version 21 (SPSS, Chicago, IL, USA). Pearson product moment was used for bivariate correlations. Un-paired t tests compared measurements between the groups with normal and impaired diaphragm movement. General linear modelling examined BDI as the dependent variable, using three predictor variables: (i) ipsilateral diaphragm movement; (ii) total lung capacity (TLC) to measure restricted lung inflation (% predicted);[25] and (iii) effusion volume (calculated as % of predicted TLC to correct for body size).[26] For general linear modelling, a sample size of ≥15 participants was required (≥ 5 participants per predictor). Predictors that were not significant were sequentially removed to obtain the parsimonious model. Results are presented as mean ± SD, except regression co-efficients which are expressed ± SE. Statistical significance was assumed when P<0.05. Assumption of normal distribution of data was assessed using descriptive methods (skewness, outliers, and distribution plots) and inferential statistics (Shapiro-Wilk test).

## Results

Thirty four patients required thoracoscopy for pleural effusion, with 19/34 meeting inclusion criteria and consenting to participate. Participants were aged 65 ± 12 years (15 male: 4 female) with body mass index 25.1 ± 4.1. Five participants had never smoked, two were current smokers, and 12 had previously smoked. Fourteen participants had malignant etiology of pleural effusion (9 mesothelioma, 4 lung cancer, 1 breast cancer) of whom 11 had talc insufflation pleurodesis. Of benign etiologies, two were idiopathic, one had cirrhosis, one had benign asbestos effusion, and one was caused by peri-splenic inflammatory collection. Effusion volume was 1.74 ± 1.04 L (14 right side: 5 left side). BDI was 6.0 ± 2.0. Baseline respiratory function are presented in Table 1, demonstrating restrictive ventilatory limitation, reduced diffusion capacity for carbon monoxide (DL_CO_), and reduced maximum inspiratory and expiratory pressures. Eleven participants had impaired diaphragm movement (4 paradoxical, 7 reduced).

**Table 1:** Baseline respiratory function.

Respiratory function parameter	Mean ± SD (% predicted) *(n = 19)*
FEV_1_, L	1.57 ± 0.45 (54.1)
FVC, L	2.22 ± 0.65 (56.3)
FEV_1_/FVC, %	72 ± 10 (96.2)
TLC, L	4.06 ± 0.86 (70.8)
FRC, L	2.29 ± 0.52 (74.6)
RV, L	1.72 ± 0.28 (78.2)
IC, L	1.77 ± 0.54 (66.6)
ERV, L	0.57 ± 0.32 (75.7)
DL_CO_, ml · min^-1 ·^mmHg^-1^	14.1 ± 4.6 (61.5)
K_CO_, ml · min^-1 ·^mmHg^-1.^ L^-1^	4.26 ± 0.90 (101.9)
V_A_, L	3.33 ± 0.86 (60.0)
V_A_:TLC ratio	0.813 ± 0.076 (n/a)
MIP, cms H_2_O *	58.8 ± 30.8 (60.4)
MEP, cms H_2_O *	89.3 ± 33.0 (66.3)

* *n* = 14, as Maximum Pressure was not measured in five participants.

FVC: forced vital capacity. TLC: total lung capacity. FRC: functional residual capacity. RV: residual volume. IC: inspiratory capacity. ERV: expiratory reserve volume. DL_CO_: diffusion capacity of carbon monoxide. K_CO_: carbon monoxide transfer co-efficient. V_A_: alveolar volume. MIP: maximum inspiratory pressure. MEP: maximum expiratory pressure. n/a: no reference values.

### Co-morbidities

Mild co-morbidities potentially causing dyspnoea were present in 7/19 participants. Mild airflow obstruction (FEV1/FVC between 50% and lower limit of normal) was present in five participants suggesting mild chronic obstructive pulmonary disease, but requiring no inhaled therapy. Two participants had mildly impaired left ventricular function (left ventricle ejection fraction 40–50%, with no clinical evidence of cardiac failure). Two participants did not have a numerical BDI as daily activity was compromised by musculoskeletal disease (one osteoarthritis; one generalised muscle deconditioning).

### Baseline bivariate correlations

**Fig 1:**
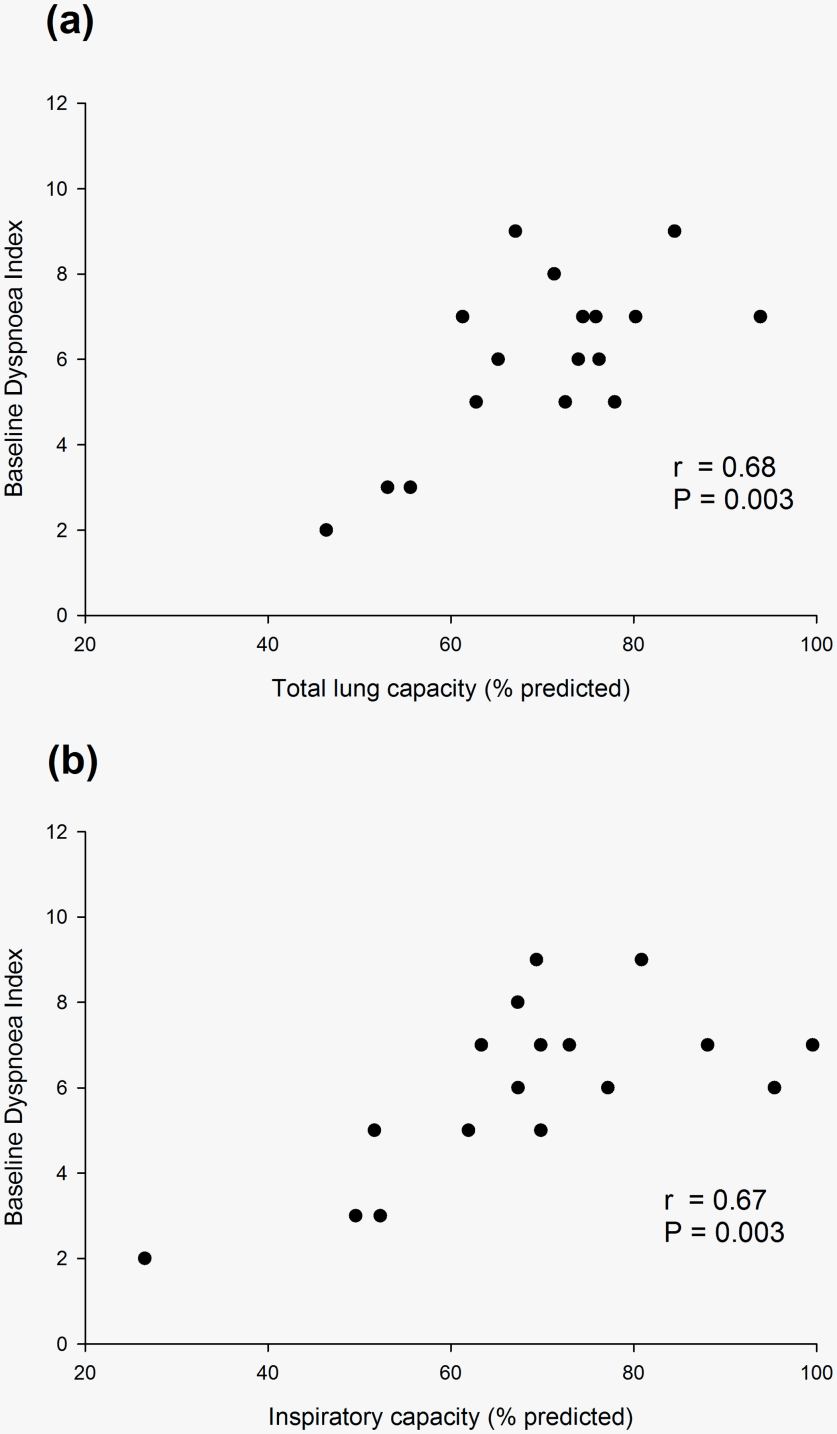
Baseline dyspnoea index (0 = most severe) significantly correlates with restricted lung inflation, as measured by (a) total lung capacity, and (b) inspiratory capacity.

The inter-observer correlation between BDI assessed by each clinician was r = 0.877. Effusion volume was not associated with BDI (r = -0.138, P = 0.60). There was a trend for BDI to be lower with abnormal diaphragm movement compared to normal diaphragm movement (5.5 ± 2.1, *n* = 11 vs. 7.0 ± 1.4, *n* = 6, P = 0.094, respectively). The most significant associations between baseline respiratory function and BDI were TLC, inspiratory capacity (IC), Alveolar Volume (V_A_) and DL_CO_ (Table 2, Fig 1). TLC was correlated with FEV_1_ (r = 0.790, P<0.001), FVC (r = 0.905, P<0.001), functional residual capacity (FRC) (0.702, P = 0.001), IC (r = 0.712, P = 0.001), expiratory reserve volume (ERV) (r = 0.616, P = 0.005), DL_CO_ (r = 0.568, P = 0.011) and V_A_ (r = 0.839, P<0.001).

**Table 2:** Correlation of baseline dyspnoea index with respiratory function.

	Entire cohort *(n = 17)*		Cohort excluding mild co-morbidity *(n = 10)*	
	r	P value	r	P value
FEV_1_, % predicted	0.452	0.069	0.797	0.006
FVC, % predicted	0.546	0.023	0.739	0.015
TLC, % predicted	0.677	0.003	0.794	0.006
FRC, % predicted	0.375	0.138	0.327	0.357
RV, % predicted	0.045	0.863	0.118	0.745
IC, % predicted	0.674	0.003	0.776	0.008
ERV, % predicted	0.300	0.242	0.225	0.532
DL_CO_, % predicted	0.607	0.010	0.808	0.005
K_CO_, % predicted	0.273	0.289	0.296	0.406
V_A_, % predicted	0.617	0.008	0.790	0.007
V_A_:TLC ratio	0.119	0.650	0.456	0.185
MIP, % predicted	-0.270*	0.396	0.096^†^	0.838
MEP, % predicted	0.049*	0.881	0.251^†^	0.587

* *n* = 12 for the entire cohort, as maximum pressure was not measured in five participants.

^†^ *n* = 7 for the cohort excluding mild co-morbidity, as maximum pressure was not measured in three participants.

Bivariate correlations were repeated after excluding seven participants with mild co-morbidity. Correlations between BDI and respiratory function were comparable to the larger cohort (Table 2) and the correlation between BDI and effusion volume remained not significant (r = -0.246, P = 0.494).

Effusion volume was larger in participants with impaired diaphragm movement compared to normal diaphragm movement (2.16 ± 0.95 L, *n* = 11 vs.1.16 ± 0.92 L, *n* = 8, P = 0.009, respectively). TLC, FEV_1_, FVC, ERV, V_A_ and V_A_:TLC ratio were all significantly lower with impaired diaphragm movement, compared to normal diaphragm movement (Table 3).

**Table 3:** Comparison of baseline respiratory function between participants with normal and impaired diaphragm movement.

Respiratory function parameter (Mean ± SD)	Normal diaphragm movement (*n* = 8)	Impaired diaphragm movement (*n* = 11)	P value
FEV_1_, % predicted	67.8 ± 15.8	44.2 ± 8.6	0.003
FVC, % predicted	68.4 ± 12.1	47.6 ± 10.0	0.001
TLC, % predicted	78.2 ± 8.6	65.4 ± 10.3	0.011
FRC, % predicted	82.8 ± 15.4	68.7 ± 12.3	0.051
RV, % predicted	76.9 ± 9.7	79.1 ± 9.3	0.625
IC, % predicted	73.0 ± 17.6	61.8 ± 17.3	0.185
ERV, % predicted	112.4 ± 57.4	49.0 ± 28.7	0.006
DL_CO_, % predicted	70.1 ± 24.0	55.2 ± 12.8	0.141
K_CO_, % predicted	97.6 ± 27.1	105.0 ± 15.2	0.498
V_A_, % predicted	70.8 ± 6.4	52.2 ± 8.9	<0.001
V_A_:TLC ratio	0.87 ± 0.06	0.77 ± 0.06	0.004
MIP, % predicted *	74.1 ± 32.7	50.0 ± 20.7	0.152
MEP, % predicted *	71.9 ± 22.5*	62.2 ± 18.5	0.410

* *n* = 6 for the group with normal diaphragm movement, as maximum pressure was not measured in two participants. *n* = 8 for the group with impaired diaphragm movement, as maximum pressure was not measured in three participants.

Effusion volume was not significantly correlated with respiratory function parameters, with the exception of FRC (r = -0.520, P = 0.022) and ERV (r = -0.576, P = 0.010). A linear model which used effusion volume (as % of predicted TLC) to predict TLC (% predicted) had intercept 77.8 ± 5.0% predicted and slope -0.23 ± 0.14 (P = 0.13).

### Baseline multivariate predictive model

A model using TLC alone was the most parsimonious model to predict BDI (adjusted R^2^ = 0.422, P = 0.003). The intercept was -1.96 ± 2.27 (P = 0.40), and the co-efficient for slope was 0.113 ± 0.032% predicted TLC^-1^ (P = 0.003). The prediction of BDI was not significantly improved by the most complex model using three predictors (TLC, effusion volume and diaphragm movement; adjusted R^2^ = 0.345, P = 0.037), or a model using two predictors (TLC and effusion volume; adjusted R^2^ = 0.390, P = 0.012).

### Bivariate correlations with improvement in dyspnoea after pleural effusion was controlled

One participant died one week after thoracoscopy. A further three participants declined assessment of TDI. Of the remaining 15 participants, nine had pleural effusion controlled at both 4 and 8 weeks.

When effusion was controlled, TDI did not change between 4 and 8 weeks (4.8 ± 2.5 vs. 4.4 ± 3.4, P = 0.805, respectively). The average TDI between 4 and 8 weeks was examined in bivariate correlation. Baseline effusion volume was the most significant predictor of TDI (r = 0.775, P = 0.014). The only significant associations between baseline respiratory function and TDI were for V_A_ (r = -0.742, P = 0.022), FEV1 (r = -0.693, P = 0.038) and TLC (r = -0.690, P = 0.040). BDI was not significantly associated with TDI (r = -0.568, P = 0.142). Although there were insufficient participants for statistical analysis, TDI appeared to improve more with impaired baseline diaphragm movement compared to normal diaphragm movement (7.1 ± 1.0, *n = 4* vs. 3.0 ± 2.2, *n = 5*, respectively).

## Discussion

Prior to drainage of unilateral pleural effusion, restricted lung inflation was associated with increased impact of dyspnoea on daily activity. Patients with impaired ipsilateral diaphragm movement prior to drainage had larger effusions and more restricted lung inflation. There was no independent additional association between dyspnoea and either effusion volume or impaired ipsilateral diaphragm movement, after accounting for restricted lung inflation. A secondary observation in nine participants was that larger effusion volume and more restricted lung inflation at baseline predicted greater improvement in dyspnoea after drainage and sustained control of effusion.

We observed significant associations between dyspnoea and a series of lung volume parameters (TLC, IC, V_A_, FEV_1_ and FVC) which are each reduced in restrictive lung disease.[25] TLC explained 46% of the variability in BDI, indicating a moderate association between more restricted lung inflation and the impact of dyspnoea on daily activities. We are not aware of other published studies examining associations between dyspnoea and respiratory function in this patient population. One study reported that shorter 6 minute walk distance (which may indicate greater impairment of daily activity due to dyspnoea) was associated with lower FVC after thoracentesis, but did not report the association before thoracentesis.[27]

Patients with impaired ipsilateral diaphragm movement prior to thoracoscopy had a larger effusion, reduced TLC and FVC, and increased dyspnoea. This is in agreement with a study which observed more severe dyspnoea and lower FVC in patients with paradoxical ipsilateral diaphragm movement, compared to patients with normal diaphragm movement.[11] There are several potential explanations for the association between restricted lung inflation and impaired diaphragm movement. The mass effect of an effusion to impair diaphragm movement may directly impair the diaphragmatic contribution to lung inflation.[3] However, it is also possible that both the impaired diaphragm movement and reduced TLC are caused by an increased mass effect of the effusion (on the diaphragm and lung respectively). Consistent with this notion, we observed that pleural effusion was on average 86% larger with impaired diaphragm movement, compared to normal diaphragm movement. Although impaired diaphragm movement was associated with restricted lung inflation, there was no independent additional association between dyspnoea and impaired diaphragm movement, after accounting for the association between dyspnoea and restricted lung inflation.

Impaired diaphragm movement was also associated with reduced V_A_:TLC ratio. A larger pleural effusion may cause wasted cross-ventilation between the two lungs due to paradoxical (superior) ipsilateral diaphragm movement on inspiration.[11, 23, 24] Such cross-ventilation would impair distribution of the dilutional gas used to measure V_A_ which may explain the reduced V_A_:TLC ratio.

In the present study, TLC and most other respiratory function parameters were not significantly associated with effusion volume; this may be due to sample size. Previous research has shown that variability in the relative compliance of the lung and chest wall contributes to variability of TLC for a given effusion volume.[5] Variability of underlying pathology causing the effusion also potentially contributes to variability of TLC for a given effusion volume. A high proportion of participants had malignancy, and ~30% of patients with malignant pleural effusion have incomplete lung expansion after chest tube drainage of effusion.[28] It must be emphasised that this cohort of patients commonly had lung pathology associated with their malignancy in addition to pleural effusion. Such pathology may have modified both respiratory function and dyspnoea independently of the pleural effusion volume. We did not aim to determine the independent effect of effusion volume on respiratory function, as our methods did not allow separate assessment of the effects of pleural effusion volume and lung pathology on respiratory function.

The lack of a significant association between dyspnoea and effusion volume was unexpected, although we are not aware of other published studies examining this association. Dyspnoea caused by restrictive lung disease is a complex phenomenon, but is likely related to an imbalance between impaired ventilatory mechanics (reduced respiratory system compliance) and inadequate inspiratory muscle function.[29–31] A larger pleural effusion reduces respiratory system compliance [32] and also displaces the thoracic cage which impairs inspiratory muscle function.[7] Respiratory compliance and inspiratory muscle function may be variably reduced for a given effusion volume, but also variably reduced by the associated pathology causing the effusion. We can conclude that the lack of any significant association between effusion volume and dyspnoea prior to thoracoscopy was potentially due to sample size. However, restricted lung inflation was more closely associated with dyspnoea than effusion volume.

Larger baseline effusion volume was associated with greater improvement in dyspnoea after pleural effusion was drained and controlled for eight weeks. Consistent with this finding, others have observed that greater improvement in dyspnoea within 24 hours of a drainage procedure was associated with the volume of fluid drained.[10] Greater improvement in dyspnoea after control of effusion was also associated with lower TLC before drainage. It can be hypothesised that restricted lung inflation (measured by TLC) may be due to combined effects of pleural disease to reduce both respiratory compliance and inspiratory muscle function. This potentially explains a greater improvement in dyspnoea after an effusion associated with restricted lung inflation is controlled, when adverse effects of the effusion on respiratory compliance and inspiratory muscle function are ameliorated.

Significant study limitations were heterogeneity in both the causes of pleural effusion and therapeutic interventions—such as talc pleurodesis—may also have modified dyspnea, but the independent association between these potential clinical predictors and dyspnea was not assessed. The general linear model for dyspnoea only had ≈ 6 participants per independent variable predictor, and therefore may have been under-powered. Our conclusions therefore require replication in a bigger cohort. We used the previously published method to qualitatively categorise diaphragm movement as normal or abnormal. However, it would be helpful for future research to develop a continuous measurement of diaphragm movement, as this should enable more accurate delineation of the relationships between diaphragm movement and the related variables we have studied. There were insufficient participants with sustained control of effusion to examine multivariate analysis of predictors of improvement in dypnoea, which was a secondary objective. Some participants had mild co-morbidities which potentially modified dyspnoea. However, mild co-morbidity did not appear to modify the association between dyspnoea and either respiratory function or effusion volume.

Prior to drainage of unilateral pleural effusion, restricted lung inflation was associated with increased impact of dyspnoea during daily activity. There was no independent additional association between dyspnoea and either effusion volume or impaired ipsilateral diaphragm movement, after accounting for the associations with restricted lung inflation. Restricted lung inflation may also predict greater improvement in dyspnoea after sustained control of effusion. Restricted lung inflation may therefore be an important determinant of the dyspnoea associated with pleural effusion. This provides a basis for a hypothesis that restricted lung inflation associated with pleural effusion may be a therapeutic target to improve dyspnoea.

## Supporting information

S1 Checklist Strobe Checklist. (DOCX)

S1 Data Repository. (XLSX)

## Abbreviations

BDI: baseline dyspnoea index
DL_CO_: diffusion capacity of carbon monoxide
ERV: expiratory reserve volume
FRC: functional residual capacity
FVC: forced vital capacity
IC: inspiratory capacity
K_CO_: carbon monoxide transfer co-efficient
MEP: maximum expiratory pressure
MIP: maximum inspiratory pressure
RV: residual volume
TDI: transitional dyspnoea index
TLC: total lung capacity
V_A_: alveolar volume
