# Supplementary material for: In patients with unilateral pleural effusion, restricted lung inflation is the principal predictor of increased dyspnoea
Source: PLoS One. 2018 Oct 3;13(10):e0202621. doi: 10.1371/journal.pone.0202621 (PMC6169850; doi:10.1371/journal.pone.0202621)
Supplement: S1 Checklist — (DOCX) [file pone.0202621.s001.docx]

STROBE Statement—Checklist of items that should be included in reports of ***cohort studies***

|  | Item No | Recommendation |
| --- | --- | --- |
| **Title and abstract** | 1 | Indicate the study’s design with a commonly used term in the title or the abstract  “A cohort of patients” added to abstract (see line ) |
|  |  | Provide in the abstract an informative and balanced summary of what was done and what was found  Completed |
| Introduction | | |
| Background/rationale | 2 | Explain the scientific background and rationale for the investigation being reported  Completed |
| Objectives | 3 | State specific objectives, including any prespecified hypotheses  Completed |
| Methods | | |
| Study design | 4 | Present key elements of study design early in the paper  Completed |
| Setting | 5 | Describe the setting, locations, and relevant dates, including periods of recruitment, exposure, follow-up, and data collection  Completed |
| Participants | 6 | Give the eligibility criteria, and the sources and methods of selection of participants. Describe methods of follow-up  Completed |
|  |  | (*b*) For matched studies, give matching criteria and number of exposed and unexposed  Not relevant |
| Variables | 7 | Clearly define all outcomes, exposures, predictors, potential confounders, and effect modifiers. Give diagnostic criteria, if applicable |
| Data sources/ measurement | 8* | For each variable of interest, give sources of data and details of methods of assessment (measurement). Describe comparability of assessment methods if there is more than one group  Completed |
| Bias | 9 | Describe any efforts to address potential sources of bias  Completed |
| Study size | 10 | Explain how the study size was arrived at  Completed |
| Quantitative variables | 11 | Explain how quantitative variables were handled in the analyses. If applicable, describe which groupings were chosen and why  Completed |
| Statistical methods | 12 | Describe all statistical methods, including those used to control for confounding  Completed |
|  |  | (*b*) Describe any methods used to examine subgroups and interactions  Completed |
|  |  | (*c*) Explain how missing data were addressed  Completed |
|  |  | (*d*) If applicable, explain how loss to follow-up was addressed  Completed |
|  |  | (*e*) Describe any sensitivity analyses  Completed |
| Results | | |
| Participants | 13* | (a) Report numbers of individuals at each stage of study—eg numbers potentially eligible, examined for eligibility, confirmed eligible, included in the study, completing follow-up, and analysed  Completed |
|  |  | (b) Give reasons for non-participation at each stage  Completed |
|  |  | (c) Consider use of a flow diagram  Considered un-necessary |
| Descriptive data | 14* | (a) Give characteristics of study participants (eg demographic, clinical, social) and information on exposures and potential confounders  Completed |
|  |  | (b) Indicate number of participants with missing data for each variable of interest  Completed |
|  |  | (c) Summarise follow-up time (eg, average and total amount)  Completed |
| Outcome data | 15* | Report numbers of outcome events or summary measures over time  Completed |
| Main results | 16 | (*a*) Give unadjusted estimates and, if applicable, confounder-adjusted estimates and their precision (eg, 95% confidence interval). Make clear which confounders were adjusted for and why they were included  Completed |
|  |  | (*b*) Report category boundaries when continuous variables were categorized  Not applicable |
|  |  | (*c*) If relevant, consider translating estimates of relative risk into absolute risk for a meaningful time period  Not applicable |
| Other analyses | 17 | Report other analyses done—eg analyses of subgroups and interactions, and sensitivity analyses  Completed |
| Discussion | | |
| Key results | 18 | Summarise key results with reference to study objectives  Completed |
| Limitations | 19 | Discuss limitations of the study, taking into account sources of potential bias or imprecision. Discuss both direction and magnitude of any potential bias  Completed |
| Interpretation | 20 | Give a cautious overall interpretation of results considering objectives, limitations, multiplicity of analyses, results from similar studies, and other relevant evidence  Completed |
| Generalisability | 21 | Discuss the generalisability (external validity) of the study results  Completed |
| Other information | | |
| Funding | 22 | Give the source of funding and the role of the funders for the present study and, if applicable, for the original study on which the present article is based  Completed |

*Give information separately for exposed and unexposed groups.

**Note:** An Explanation and Elaboration article discusses each checklist item and gives methodological background and published examples of transparent reporting. The STROBE checklist is best used in conjunction with this article (freely available on the Web sites of PLoS Medicine at http://www.plosmedicine.org/, Annals of Internal Medicine at http://www.annals.org/, and Epidemiology at http://www.epidem.com/). Information on the STROBE Initiative is available at http://www.strobe-statement.org.
